# pH-regulated single cell migration

**DOI:** 10.1007/s00424-024-02907-2

**Published:** 2024-01-12

**Authors:** Christian Stock

**Affiliations:** Department of Gastroenterology, Hepatology, Infectiology & Endocrinology, Hannover Medical School, Carl-Neuberg-Str. 1, 30625 Hannover, Germany

**Keywords:** Acid priming, Acid selection, Cell migration, Molecular pH sensors, pH nanodomains, pH taxis

## Abstract

Over the last two decades, extra- and intracellular pH have emerged as fundamental regulators of cell motility. Fundamental physiological and pathological processes relying on appropriate cell migration, such as embryonic development, wound healing, and a proper immune defense on the one hand, and autoimmune diseases, metastatic cancer, and the progression of certain parasitic diseases on the other, depend on surrounding pH. In addition, migrating single cells create their own localized pH nanodomains at their surface and in the cytosol. By this means, the migrating cells locally modulate their adhesion to, and the re-arrangement and digestion of, the extracellular matrix. At the same time, the cytosolic nanodomains tune cytoskeletal dynamics along the direction of movement resulting in concerted lamellipodia protrusion and rear end retraction. Extracellular pH gradients as found in wounds, inflamed tissues, or the periphery of tumors stimulate directed cell migration, and long-term exposure to acidic conditions can engender a more migratory and invasive phenotype persisting for hours up to several generations of cells after they have left the acidic milieu. In the present review, the different variants of pH-dependent single cell migration are described. The underlying pH-dependent molecular mechanisms such as conformational changes of adhesion molecules, matrix protease activity, actin (de-)polymerization, and signaling events are explained, and molecular pH sensors stimulated by H^+^ signaling are presented.

## Introduction

Cell migration is a central component of embryonic development, wound healing, tissue homeostasis, and a proper immune defense. However, aberrant cell migration contributes to various pathologies, such as metastatic cancer and autoimmune diseases. In addition, a number of parasitic protozoa can migrate through certain tissues of the human body, particularly amoebae like the *Entamoeba histolytica* [79] or the brain-eating *Naegleria fowleri* [58]. The process of lamellipodium-driven single cell migration, whether directed or random, entails a set of requirements. A migrating cell needs to (i) polarize in the direction of movement, (ii) fine-tune its attachment to and detachment from the substrate such as extracellular matrix (ECM) components or surrounding cells, (iii) exert (traction) forces on the substrate, and (iv) reorganize or even remove parts of the surrounding ECM, especially when invading a basement membrane. Each of these mechanisms is one way or another related to, if not even directly dependent on, the pH value. While in the healing skin the migration of both fibroblasts and keratinocytes is most efficient at rather physiological pH values of around 7.5 [78], moderately acidic environments as found in tumor tissue or at inflammatory sites promote motility of tumor cells [121, 155] and neutrophils [111], respectively. Aside from that, extracellular pH gradients direct migrating cells [114, 166], and long-term exposure to relatively acidic pH values can cause cells to adopt a more migratory and invasive phenotype that can persist for hours up to several generations of cells after they have left the acidic milieu [123, 163]. The present review deals with all of these pH-sensitive parameters involved in cell migration. Underlying molecular mechanisms are explained and critically examined.

## Extracellular pH_e_ as a clue inducing cell polarity

Epithelial cells forming a single-layer epithelium feature a highly pronounced apical-basal polarity with the apical membranes facing lumina of internal cavities and the basolateral membranes being orientated away from these lumina towards the underlying tissue. A functional dichotomy accompanies this morphological polarity. Different sets of membrane proteins such as ion channels and transporters, receptors, and adhesion molecules are expressed in the apical and the basolateral membranes, in order to enable epithelia to fulfill their main physiological functions in addition to being a protective barrier between different compartments, namely secretion and absorption. Tight junctions, strong linkages between adjacent epithelial cells, keep these membrane proteins apart in the different membrane sections. In addition, regulated membrane trafficking directs the proteins to their final destination where a number of them are kept in place by adaptor proteins, such as members of the ezrin, radixin, and moesin (ERM) family that tie membrane proteins to the cortical actin cytoskeleton. Generally, the polarity of epithelial cells is predetermined by the given tissue structure and coordinated by the activity and localization of apical-basolateral polarity regulators, e.g., the PDZ-rich scaffold protein Scribble, the PDZ domain–containing adaptors Par3 and Par 6, the PDZ-containing scaffold PATJ, or the transmembrane protein Crumb 3 [99]. These cell polarity–regulating proteins act through molecular mechanisms such as oligomerization, higher-order complex formation, auto-inhibitory interactions, or electrostatic interactions with the plasma membrane [125]. However, as soon as tightly connected, epithelial cells set out to break out of the epithelial order, e.g., during the process of epithelial-to-mesenchymal transition (EMT), they lose their apical-basolateral polarity due to a fundamental reorganization of the cytoskeleton and a loss of cell–cell junctions. These structural changes come along with a profound redistribution of membrane proteins such as ion channels and transporters and receptors. A new, morphologically and functionally different polarity, a front-back polarity, forms, allowing the cells to acquire a migratory phenotype. Not only epithelial cells undergoing EMT, but also migrating cells of any other origin, including endothelial cells, fibroblasts, mesenchymal cells, immune cells, and osteoblasts/-clasts, regardless of their initial polarization status, establish a front-back polarity when they start to migrate, i.e., a morphological and functional polarity along the direction of movement [116]. As the leading edge of a migrating cell protrudes forward, the trailing end retracts.

Cues that trigger single cell polarization can be chemical gradients, mechanical stimuli, membrane tension, substrate rigidity, and electric fields [120]. The amoeba *Dictyostelium discoideum*, commonly referred to as slime mold, is a rewarding model system for investigating the mechanisms of directed amoeboid movement including the different modes of polarization. Exposure to its adequate chemoattractant cAMP induces a polarized, elongated morphology. In a fluid flow without a chemical stimulus, *Dictyostelium* orientates itself along the current, the leading edge pointing upstream, and the retracting tail downstream [28]; and in an electric field, the leading edge is directed towards the electron-emitting cathode [144]. In contrast to the external, spatial cue–driven polarization, spontaneous polarization is based on the cell’s intrinsic ability to break symmetry. Positive feedback loops involving the lipid phosphatidylinositol-3,4,5-trisphosphate and/or the small Rho-type GTPase Cdc42 are sufficient to drive spontaneous polarization [96, 182].

**Fig. 1:**
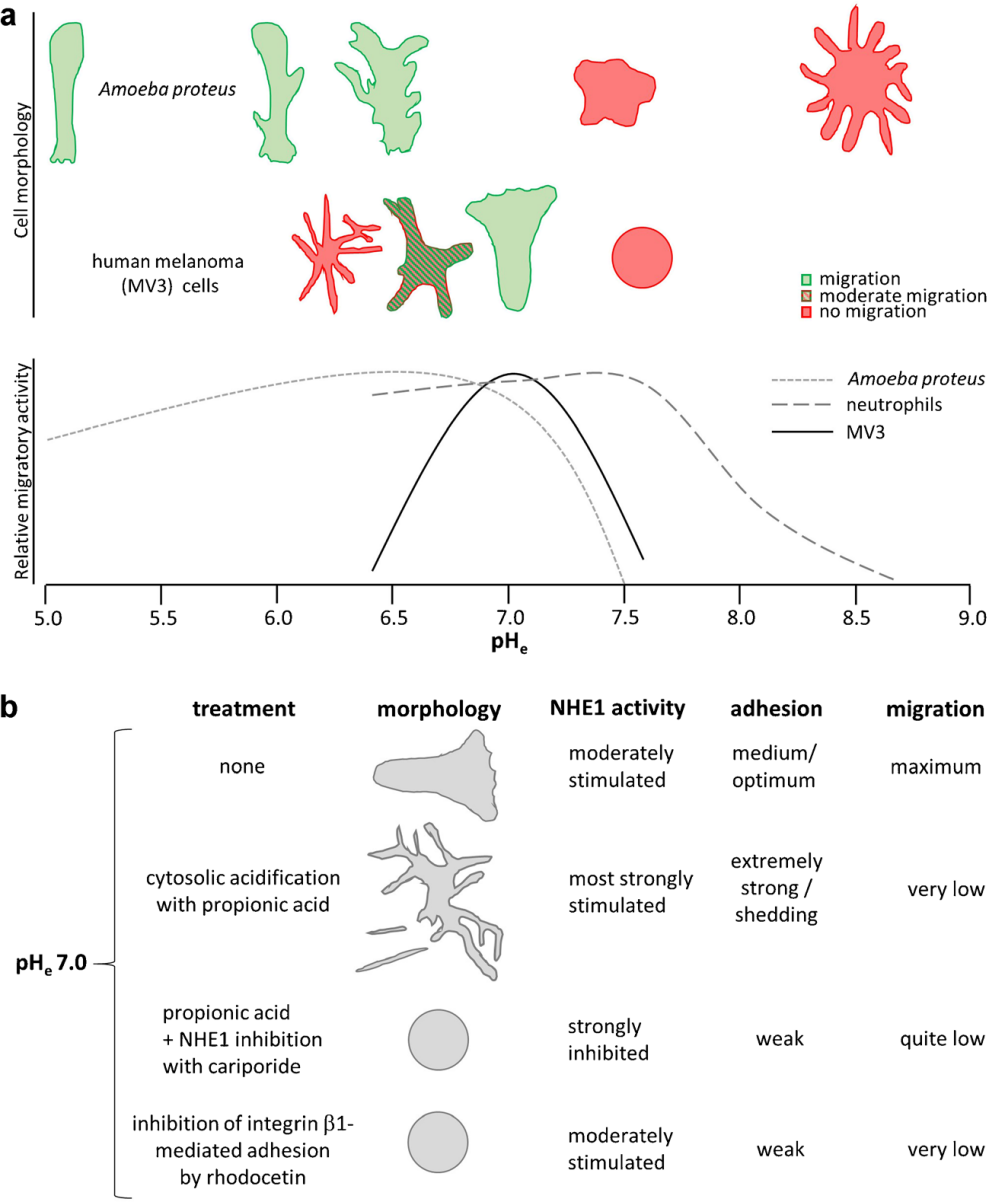
Cell polarization and migration depend on extracellular pH (pH_e_) and acid extrusion. **a** While *Amoeba proteus* shows perfect polarization and high migratory activity in a rather acid pH_e_ range of between pH 5.0 and 7.0 (11), human melanoma (MV3) cells polarize and migrate in an optimum way at pH_e_ 6.8–7.2 [155]. Neutrophils can cope with quite a wide pH_e_ range from values below 6.0 to more than 8.0 [148]. **b** Stimulation of NHE1 by cytosolic acidification with propionic acid at pH_e_ 7.0 leads to the formation of numerous sticky protrusions accompanied by a strongly inhibited migration. Inhibition of the propionic acid–stimulated NHE1 activity by cariporide causes a drastic change in cell morphology from branched to spherical. Blocking integrin α_2_β_1_ with rhodocetin at unaffected NHE1 activity and pH_e_ 7.0 results in a spherical cell shape as well [155]. These observations point to a role of both NHE1 activity and integrin dimers in cell adhesion and migration

In the end, however, in the absence of any spatial cue or chemical substance, the omnipresent parameter affecting polarity of single cells is the environmental pH value (Fig. 1a). *Amoeba proteus* exhibits the most polarized shape, accompanied by the highest migratory activity, at extracellular pH (pH_e_) values of between pH 5.0 and pH 6.5 (11). Similarly, fMLP (N-formyl-methionyl-leucyl-phenylalanine)–stimulated neutrophils migrate most efficiently at pH_e_ 7.4–7.6 [148], and also human melanoma (MV3) cells seeded on, or embedded in, a collagen type I matrix show the highest migration speeds when most perfectly polarized at pH_e_ values of 7.0–7.2 [155]. Intriguingly, cells equipped with efficient acid/base transporters in their plasma membrane, e.g., NHE1, can countervail the impact of the environmental pH [161] by generating their own pH nanoenvironment right at the cell surface [157], stabilized by the glycocalyx [77].

## Interrelations between cell polarity and pH

Regardless of whether induced or spontaneous, the polarization of migrating cells is accompanied by an accumulation of a multitude of ion transporters and channels at their leading edge [156]. These include acid/base transporters such as Na^+^-HCO_3_^−^ cotransporters (NBCs) (NBCn1 [9, 143] or NBCe1 [164]), anion exchangers (AEs) [72, 164], NHE1 [157], carbonic anhydrases [164], and water-permeable aquaporins [86, 131]. Regarding the migratory process, this accumulation of ion transporters at the leading edge serves at least two purposes.

First, osmotic water influx through aquaporins adjacent to the ion transporters is concomitant with the movement of osmotically active ions into the cytosol, which then leads to local swelling and outgrowth of the lamellipodium in the direction of movement [87, 108, 139, 150]. At the same time, the retraction of the rear part comes along with osmotic shrinkage, triggered by (membrane) stretch-activated, mechanosensitive Ca^2+^-channels [18, 108, 156], and mediated by Ca^2+^-sensitive K^+^ [137, 138, 142], Cl^−^ [134], and water efflux, the latter most likely facilitated by aquaporins [129].

Second, in parallel with this “osmotic engine,” driven by the directed movement of ions and water across the cytosol from the leading edge to the trailing end [160], migrating cells utilize the accumulation of pH regulatory transporters at the leading edge to establish pH gradients along the direction of movement [158]. The Na^+^/H^+^ exchanger NHE1 (*SLC9A1*) accumulates at the leading edge of migrating fibroblasts (human and hamster lung [48]; murine embryonic [95]), renal epithelial cells (canine [72]), and melanoma cells (human MV3 [157] and murine B16V cells [95]). In serum-starved, quiescent cervical cancer cells, the epidermal growth factor (EGF) triggers polarization and subsequent migration by inducing a redistribution of randomly distributed NHE1 to the simultaneously developing lamellipodia [20]. There, at the leading edge, the H^+^-extruding activity of NHE1 causes a local acidification of the cell surface [157] and a complementary cytosolic alkalinization of the lamellipodium [95]. Regardless of whether exposed to a HCO_3_^−^/CO_2_ or a HEPES (2-[4-[2-hydroxyethyl]piperazin-1-yl]ethanesulfonic acid)–buffered medium, the cytosolic pH difference between the more alkaline lamellipodium and the more acidic trailing end comes to a ΔpH of 0.15 in human and murine melanoma cells (MV3, B16V). In contrast, it is clearly lower (ΔpH ~ 0.05) in non-malignant cells such as fibroblasts (NIH3T3) and endothelial-like (EA.hy926) cells [95]. This considerable difference in the front-back ΔpH_i_ between malignant and non-malignant cells is consistent with the general upregulation of net-acid extruding transporters in metabolically highly active cancer cells compared to normal cells [42]. Accordingly, and consistent with a certain resistance to the absence of HCO_3_^−^, MV3 and B16V cells show significantly higher NHE1 activity at their lamellipodia than NIH3T3 fibroblasts, resulting in a steeper intracellular pH gradient along the direction of movement [95]. In MV3 cells, the intracellular pH gradient is mirrored by a cell surface pH gradient with a ΔpH of up to 0.2 between the more acidic leading edge and the rear end [157]. The pH gradient at the cell surface is maintained by the cell’s intact glycocalyx. Removing N-glycosides from the glycocalyx, either with tunicamycin that suppresses the formation of N-glycosidic linkages in the endoplasmic reticulum or by a mixture of glucosaminidase and PNGaseF (peptide-N4-(N-acetyl-beta-glucosaminyl) asparagine amidase) that remove almost all N-linked oligosaccharides from the cell surface glycoproteins, leads to both a collapse of the cell surface pH gradient and a significant decrease in the migratory activity [77]. Upon stimulating NHE1 activity by cytosolic acidification with propionic acid at unchanged extracellular pH, glycocalyx-deficient cells re-establish their cell surface pH gradient, but with considerably lower pH values, and regain their ability to migrate [77]. These observations suggest that the glycocalyx acts as a diffusion barrier for protons, especially laterally, with the aim of creating locally defined pH domains at the cell surface, which then ensure both the maintenance of cell polarity and a smooth migration.

In fact, in addition to the existence of the cell surface pH gradient, cell migration requires the presence of cell adhesion molecules (CAMs) such as cadherins, selectins, or integrins. In MV3 cells, integrin α_2_β_1_ dimers mediate adhesion to, and migration on, a collagen type I substrate [93]. Neither the mere availability of intact α_2_β_1_ integrin dimers at the cell surface in the absence of the longitudinal cell surface pH gradient nor the presence of the pH gradient alone, in the absence of intact α_2_β_1_ integrins in β_1_-deficient cells, is sufficient to enable MV3 cells to migrate [77]. These findings imply an interdependence between adhesion (forces) and pericellular pH, particularly with regard to the fact that integrins with extended conformation protrude from the surface of the plasma membrane by ~ 20 nm [107].

## Extracellular pH nanodomains locally modulate essential components of the migratory machinery

### Cell-matrix adhesion

**Fig. 2:**
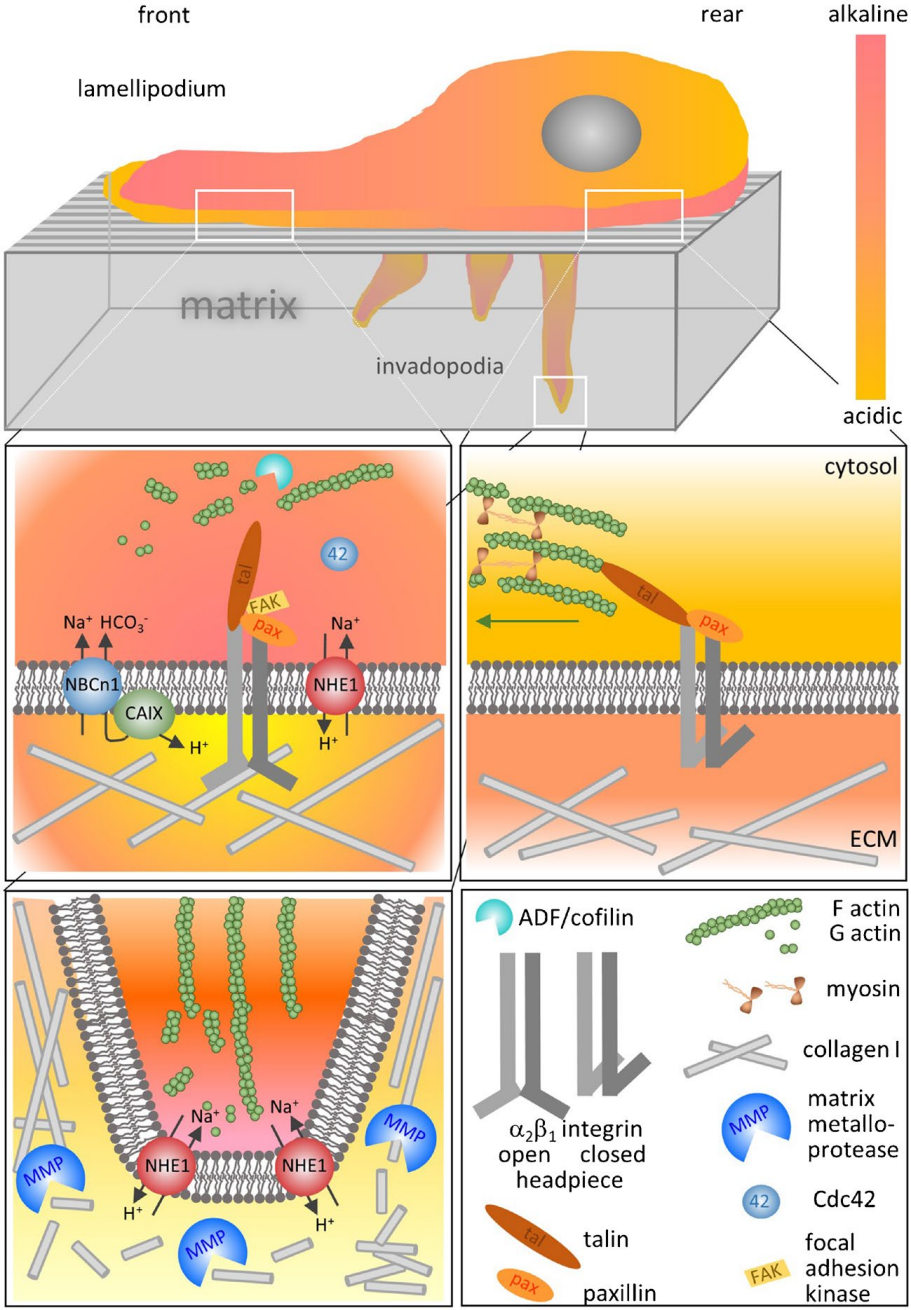
pH gradients and pH nanodomains in the cytosol and at the cell surface modulate focal adhesion dynamics, migration, and invasion. NHE1 and other acid/base regulators such as NBCn1 accumulate around focal adhesions at the leading edge where they locally (i) alkalinize the submembranous zone of the cytosol and (ii) acidify the cell surface. Membrane-bound carbonic anhydrases such as CAIX physically interact with NBCn1 and supply the HCO_3_^−^ to be transported into the cell. Alkaline pH_i_ values reduce actin binding by talin and stimulate the activities of FAK, Cdc42, and the actin-severing protein cofilin, leading to increased actin and focal adhesion dynamics. Acidic pH_e_ values increase integrin avidity and promote the formation of integrin-matrix bonds. At the cell rear, an alkaline pH_e_ weakens adhesion, while an acidic pH_i_ (i) slows down actin and focal adhesion dynamics due to reduced cofilin and FAK activity, (ii) strengthens actin-talin binding, and (iii) stimulates myosin activity, which then jointly promotes the retraction process. At the tips of invadopodial structures, NHE1 activity acidifies the surface and thus provides optimum pH conditions for ECM-degrading MMPs that clear the way. Please see text for further details and references

The interaction forces between integrin dimers at the cell surface of osteoclasts and their Arg-Gly-Asp peptide (RGD) sequence containing ligands, such as fibronectin and vitronectin, are strongly pH-dependent, with the highest binding force at pH 6.5 [81]. As shown for integrin α_v_β_3_, extracellular pH modulates the conformation of integrin dimers and thus regulates their activity, including the avidity between the integrin dimer and an ECM molecule [113]. In migrating MV3 cells, α_2_β_1_ integrins and NHE1 colocalize at focal adhesion sites of the outgrowing lamellipodium, where NHE1 activity creates a locally acidic nanoenvironment at the cell surface (Fig. 2). Pericellular pH, including the more acidic pH nanodomains at focal contacts identified by DsRed2-paxillin, was determined by ratiometric fluorescence measurements using total internal reflection fluorescence (TIRF) microscopy, after either the N-glycosidic linkages of the glycocalyx or the outer leaflet of the plasma membrane had been labeled with the pH-sensitive fluorescein-conjugates WGA (wheat germ agglutinin) and DHPE (1,2-dihexadecanoyl-sn-glycero-3-phosphoethanolamine), respectively [77, 90, 157]. This local acidification then supports the formation and possibly the maturation of focal adhesions, most likely by unfolding the headpieces of the α_2_β_1_ integrin dimers, allowing a strong interaction with ECM proteins [90]. The accumulation of hypoxia-induced, cell surface-bound carbonic anhydrase IX (CAIX) at both nascent and maturing focal adhesions strongly suggests its assisting role in the generation of acidic nanodomains at the cell surface and possibly alkaline nanodomains in the cytosol [26]. Becker and Deitmer [6] advanced the more than 20-year-old concept of carbonic anhydrase–associated transport metabolons [122, 153]. Transport metabolons are structural and functional complexes that reside in the plasma membrane and consist of a carbonic anhydrase such as CAIX and a HCO_3_^−^ or H^+^ transporter, e.g., AEs, NBCs, NHEs, or monocarboxylate transporters (MCTs). Especially in metabolically highly active tumor cells under hypoxic conditions, glycolytic metabolites and H^+^ ions accumulate. Becker and Deitmer [6] propose that intracellular CAII directly bound to the C-terminal tail of MCT and CAIX bound to the MCT chaperone CD147 both function as “proton antennae,” thus facilitating the rapid exchange of H^+^ between the transporter pore and surrounding protonable residues near the cell membrane. This efficient mechanism would not only drive the export of H^+^ and lactate to allow a high glycolytic rate but could also modulate proton-sensitive interactions between cell surface and ECM.

Quite recently, the presence of acidic nanodomains around focal adhesions was verified by a different experimental approach: single cells were seeded on coverslips coated with chemically immobilized pH-sensitive fluorescein isothiocyanate (FITC), and the fluorescence intensity of the substrate underneath the cells was detected by utilizing classical fluorescence microscopy. Here, focal adhesions were identified by immune-labeling vinculin, a major cytosolic component of focal contacts [98]. No matter whether identified by DsRed2-paxillin or immune-labeled vinculin, single, typically oval focal adhesions cover an area of ~ 3 µm^2^, measuring 4 µm in length and nearly 1.4 µm at their widest [69]. The areal extent of focal adhesions therefore does not allow pH domains with a radial expansion of less than ~ 1.4 µm to be measured. However, since (i) in the applied TIRF microscopy [90] the evanescent wave penetration depth at an excitation wave length of 488 nm is 80–150 nm [36], (ii) the distance between ventral cell surface and the substrate at the focal contacts is approximately 10–20 nm [15], and (iii) removal of the glycocalyx leads to a disappearance of the pH domains [77], which have different pH values directly at the plasma membrane than in the glycocalyx [157], the axial expansion of the pH nanodomains is actually in the range of 10 to several 100 nm.

Once strong focal contacts are established, the cell can move over the substrate utilizing it as an opposite force to exert its own traction and compressive forces, mainly generated by actomyosin contractility [49]. Towards the trailing end, both the density and the activity of NHE1 decrease. The resulting relative alkalization around the integrins weakens the adhesion forces between cell and substrate, and eventually facilitates the disengagement of focal adhesions so that the cell can smoothly retract its rear end and move forward. This is consistent with the observation that stimulation of NHE1 activity by cytosolic acidification with propionic acid at unchanging physiological bulk pH_e_ values stimulates the formation of sticky lamellipodia-like protrusions (Fig. 1b). These protrusions are sticky to such an extent that they cannot be detached from the matrix anymore in order to be retracted. They need to be shed off the cell body instead. Conversely, specific inhibition of NHE1 with cariporide (HOE642) at physiological pH_e_ drastically reduces the cells’ adhesiveness resulting in a nearly perfectly spherical cell shape, like that found after trypsinization of cultured cells when passaging them [155, 157, 161]. Similarly, at pH_e_ 7.0 and regular NHE1 activity, the inhibition of α_2_β_1_ integrin dimers by rhodocetin, a C-type lectin–related protein isolated form the venom of the Malayan pit viper [31], causes the cells to become spherical [155], and blocks the residual, pH-dependent migratory activity in β_1_-deficient MV3 cells completely [77].

#### pH-dependent integrin-mediated outside-in signaling

By modulating number and strength of integrin bonds, pericellular pH potentially affects cell migration also by integrin-mediated outside-in signaling. Bound integrins, especially the β-subunits, signal via Src family kinases, the focal adhesion kinase (FAK), and a number of small G proteins in order to regulate PI3K-AKT, MAPK, ITAM-PLC/Ca^2+^, and RhoA-ROCK signaling pathways whose intricate interplay fine-tunes the migratory machinery [105, 145]. Following the (pH-dependent) binding of extracellular ligands to the integrin dimer, an activated subunit of a heterotrimeric G protein, Gα_13_, binds to the integrin β subunit and thus (i) enhances integrin outside-in signaling and (ii) feeds back to GPCR (G protein–coupled receptor)–stimulated RhoA activation [46, 145]. Four members of the group of GPCRs, namely GPR4, TDAG8 (GPR65), OGR1 (GPR68), and G2A (GPR132), are proton sensors [149], and at this point, it is worth mentioning that TDAG8 has been shown to act through Gα_13_/Rho signaling in blood cancer cells [61]. Hence, it is conceivable that the pericellular pH affects integrin-mediated outside-in signaling not only directly through the number and through the strength of focal adhesions but also indirectly by modulating integrin-mediated signaling via subunits of G proteins activated by proton-sensing GPCRs.

Also in astrocytes, integrin-mediated outside-in signaling leads to the activation and localized recruitment of Cdc42, a member of the Rho GTPase family. Cdc42 then generates a persistent polarity of the migrating astrocyte by promoting (i) Rac-dependent protrusion and (ii) PKCζ/dynein-dependent reorientation of the Golgi, the microtubule organization center, and the microtubule network towards the leading edge [33].

### Cell–cell adhesion

**Fig. 3:**
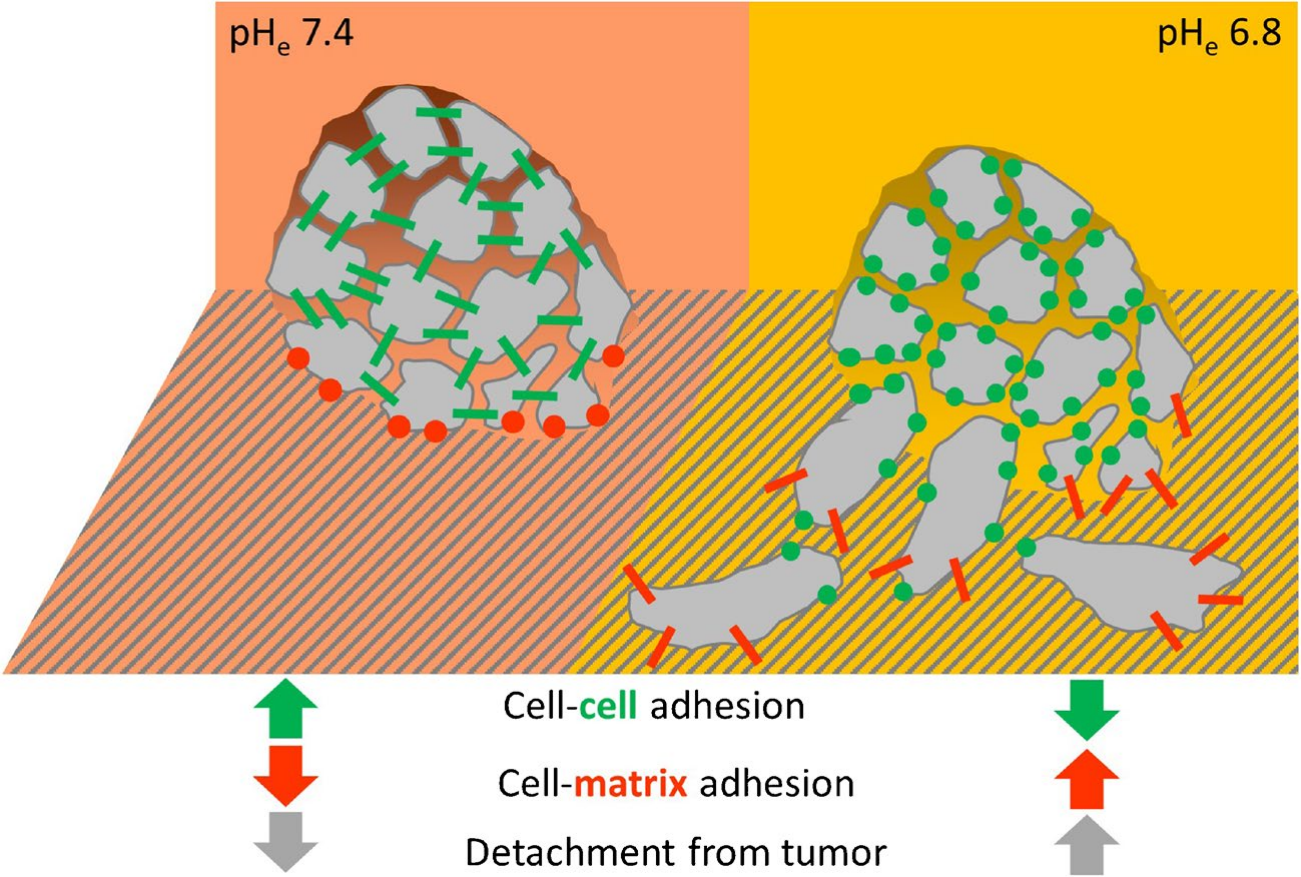
Extracellular acidification weakens cell–cell adhesion and stimulates cell–matrix adhesion at the same time. These simultaneous effects are thought to synergistically promote metastasis by facilitating both the detachment of single cells from a primary tumor and the invasion of the surrounding tissue [53]

Cell–cell interaction depends on pH_e_ and is modulated by NHE1 as well, however in opposite direction to their effects on cell–matrix interactions [53]. While acidic pH_e_ and strong NHE1 activity strengthen cell–matrix adhesion, they weaken cell–cell adhesion (Fig. 3). In view of tumor diseases, this is relevant for the metastatic process because in solid tumor tissue the interstitial pH is considerably lower than in the surrounding healthy tissue and decreases from the tumor edge to the tumor center [57]. Indeed, low cell–cell adhesion forces facilitate detachment of cells from primary melanoma (MV3) spheroids. Thus, a synergistic interplay between pH-dependent cell–matrix and cell–cell adhesions harmonizes different steps of the metastatic cascade. Protons secreted by NHE1 promote metastasis by first facilitating cell detachment from the primary tumor and subsequently modulating cell–matrix interactions to drive cell migration and invasion [53].

### Cell invasion and metastasis—activity of matrix proteases

Cell invasion is based on migration and defines the ability of cells to navigate through the ECM within a tissue or to infiltrate neighboring tissues by crossing the basement membrane that usually separates them. For an invading cell to have sufficient space, the ECM must be remodeled, if not locally digested. The task of ECM remodeling including cleavage of most ECM components is performed by matrix-degrading proteases [89]. The pH optimum for the activity of a number of ECM-degrading proteases such as cathepsins B [45], D [12], L [29], and S [13] is clearly more acidic than the physiological pH_e_ of 7.3–7.4 measured in normal and healthy tissues [44]. In addition to pure enzymatic activity, pH sensitivity of both half-life/stability and activation from inactive precursors (pro-proteases) also contributes to pH-dependent proteolytic activity of matrix proteases. Thus, a low pH_e_ favors the activity of interacting proteolytic cascades, which in turn convert pro-matrix metalloproteinases (proMMPs) into active MMPs [73]. Human stromelysin-1, also known as matrix metalloproteinase-3 (MMP3), is involved in the activation of pro-MMP1, -7, -8, and -9 [27] and exhibits its maximum activity in a narrow range of pH values ranging from pH 5.75 to 6.25 [54]. Interestingly, the activity of MMP3 relies on its own protonation, i.e., protonation of its His^224^ [54, 60], whereas the activity of MMP2 depends on the extent to which its substrate fibrinogen is protonated [106]. Not only the stability and activity, but also the expression and secretion of ECM-degrading enzymes may depend on pH_e_. Thus, an acidic environment stimulates the expression of MMP9 through calcium influx–triggered phospholipase D-mitogen-activated protein kinase signaling and acidic sphingomyelinase activation [65, 66] and induces the release of cathepsin B [130].

Because NHE1 contributes greatly to the pericellular pH nanoenvironment, it is hardly surprising that it also affects the secretion and activity of ECM-degrading proteases [16, 47, 171]. Noteworthily, stable transfection with an ion translocation-defective NHE1 reduces both gene expression and activity of MMP9 [119], and in breast cancer cells, NHE1 inhibition blocks the CD44-dependent increase in cathepsin B maturation and activity [10].

In conclusion, the pericellular pH nanoenvironment of migrating/invading cells with a more acidic pH_e_ either at the very edge of the lamellipodium or at the tips of invadopodia protruding into the ECM leads to localized pH-sensitive proteolytic activity very close to the membrane (Fig. 2). Interestingly, an integrin β_1_-mediated adhesion to ECM proteins and a pH-dependent collagen I-digesting activity of cysteine peptidases at the surface of the brain-eating amoeba *Naegleria fowleri* are assumed to play a critical role in their invasion of the central nervous system [58, 176]. At first glance, it may seem contradictory that acidification at the cell surface causes the cell to bind more strongly to the matrix, while at the same time digesting the matrix to which the cell is supposed to adhere. However, it is thought that digestion of and adhesion to the matrix do not occur at the very same spot. While the ECM at the front of the lamellipodial/invadopodial structures needs to be softened and dissolved, focal adhesion sites acting as anchors and counterforce are created and stabilized a little further back, for example, at the base of the emerging invadopodia [141, 154].

## Intracellular pH nanodomains locally modulate essential components of the migratory machinery

### Cytosolic constituents of focal adhesion complexes

As NHE1 activity acidifies adhesion foci at the cell surface, it simultaneously—and just as locally—alkalizes those submembranous regions of the cytosol that house the cytoplasmic domains of the adhesion molecules, their numerous structural and signaling interaction partners, and other proteins involved in the formation, maturation, and disassembly of focal adhesions [21, 90]. A number of these molecules react in a pH-dependent manner.

#### Talin

The adaptor protein talin, also called the master of integrin adhesions [71], links integrins directly to actin [109]. One of its several actin bindings sites is pH-dependent, with a more than twofold greater affinity of F-actin binding at pH 6.5 compared to pH 7.5 [152]. This is concomitant with a shorter lifetime of focal adhesions combined with a higher migratory rate at higher intracellular pH (pH_i_) and a decreased focal adhesion turnover accompanied by a reduced migratory activity at lower pH_i_ values [152]. The pH dependence of their mechanical stability was confirmed on isolated focal adhesions by means of bead-pulling experiments employing a magnetic microneedle apparatus [5]. Correspondingly, the cytosolic pH gradient in migrating cells with more alkaline pH_i_ values in the front region allows for higher focal adhesion turnover and actin dynamics at the cell front, supporting sampling of the surrounding substrate, actin treadmilling, and lamellipodial growth. The more acidic pH_i_ towards the trailing edge stabilizes the integrin- and talin-mediated connection between the plasma membrane and F-actin ensuring the efficient and complete retraction of the rear end. Talin’s histidine^2418^ was identified as an essential element of the pH-dependent molecular switch regulating F-actin binding [152].

#### Vinculin, paxillin, kindlin, and zyxin

Vinculin being part of the talin-vinculin axis is another major constituent of focal adhesions. It regulates and transmits mechanical forces between the cytoskeleton and adhesion receptors [101], for instance, by maintaining talin in its extended conformation [19]. However, observations on its pH dependence are contradictory [102, 112].

In addition to vinculin, paxillin, kindlin, and zyxin are typical constituents of focal adhesion complexes [62, 63]. Although a pH-dependent role of these proteins in focal adhesion assembly and cell motility has not been shown explicitly, at least paxillin and zyxin may well be pH-dependent. They are furnished with several c-terminal LIM domains that bind to mechanically stressed (tension, compression, twist-bend coupling) actin filaments [186]. The recruitment of paxillin to α_v_β_3_ integrin–positive focal adhesions also depends on LIM domains [126]. The activity of these LIM domains is likely to depend on pH. In the small flowering plant *Arabidopsis* (*Brassicaceae* family), a group of these LIM domains can affect actin-bundling in a pH-dependent manner, probably due to an accumulation of acidic amino acids at their C-termini [104].

#### Focal adhesion kinase (FAK)

FAK is a central regulator of focal adhesion remodeling. It binds to the cytoplasmic domain of integrin β subunits and transduces growth factor and integrin signals for survival [1], adhesion dynamics [180], and migration [147]. FAK is directly sensitive to physiological changes in pH. An alkaline pH causes deprotonation of FAK-His^58^ and thus drives conformational changes that modulate the accessibility of Tyr^397^ to enable its autophosphorylation [21], which is required for the subsequent Src-mediated phosphorylation of the catalytic Tyr^576^ and Tyr^577^ [17, 83]. Conversely, a substitution of His^58^ by alanine with its hydrophobic, non-reactive methyl side chain allows autophosphorylation and cell spreading at low pH_i_, too [21].

### Cytoskeletal dynamics and contractility

Cell migration relies on coordinated periodic lamellipodial protrusion and rear end retraction, accomplished by a highly dynamic regulation of actin structures at the leading edge and an appropriate actomyosin-mediated contractility at the rear end. pH_i_ controls both actin dynamics in the lamellipodium and actomyosin-mediated contractility towards the cell rear.

#### Gelsolin and actin-depolymerizing factors/cofilin

The actin-regulatory, cytoplasmic gelsolin plays a crucial role in remodeling the actin cytoskeleton during cell migration by severing, capping, and uncapping actin filaments. Its F-actin-severing activity is modulated by phosphatidylinositol-4,5-bisphosphate (PIP2) binding and requires a high (between 10^−6^ and 10^−5^ mol L^−1^) intracellular Ca^2+^ concentration [Ca^2+^]_i_ and a low pH_i_ [35, 162, 189]. The low pH_i_ leads to local structural changes caused by protonation of two histidines (His^29, 151^) and one aspartate (Asp^109^) within the first of six subunits. While protonation of all three is essential for pH-dependent actin-severing activity, protonation of His^151^ directly affects filament binding because it resides right at the gelsolin/actin interface [35].

Being a member of the actin-depolymerizing factor (ADF)/cofilin family, cofilin is another pH sensor acting on actin dynamics [38, 187]. Increased assembly of a branched actin filament network drives membrane protrusion at the leading edge of migrating cells [8, 117]. In the outgrowing lamellipodium, a zone of fast F-actin polymerization subjacent to the leading edge is followed by an area of virtually complete depolymerization of actin filaments a few micrometers back, generating a 2–4-µm treadmilling actin array adjoining the leading edge [118, 179]. Actin monomers originating from the depolymerization process recycle to the zone of polymerization to keep the treadmill running. Cofilin (i) facilitates the fragmentation of actin filaments in the depolymerization zone resulting in an increase in actin monomers, and (ii) debranches filaments to generate new free barbed ends for nucleation by the Arp2/3 complex [8]. A rather alkaline pH, as found in the front end of migrating cells due to local NHE1 activity [90, 95], leads to deprotonation of cofilin’s His^133^, thus weakening the bond between PI[4, 5]P2 in the inner leaflet of the plasma membrane and cofilin [38]. This results in the release of active cofilin from the membrane followed by increases in both actin free barbed ends and available actin monomers [64]. Similarly, in invadopodial structures as well as in lamellipodia, cortactin-bound cofilin is released at more alkaline pH due to a reduced cortactin-cofilin binding affinity, again increasing barbed end generation, while a decrease in local pH pushes cofilin to (re)bind to cortactin [94].

To emphasize the cross-species universality of pH (gradient)–dependent protrusion growth and motility mechanisms, it should be mentioned here that the growing pollen tube of *Arabidopsis thaliana* (*Brassicaceae* family) establishes a pH gradient along the direction of growth and that isovariants of actin-depolymerizing factor (ADF) with different pH sensitivity drive pollen tube growth by acting pH dependently and thus locally in different regions of the growing tube [178]. Similar observations were made earlier in growing lily pollen tubes, where the pH-sensitive ADF together with actin-interacting protein localize to the cortical actin fringe region. Acidification with sodium acetate causes actin filament destabilization in the apical and subapical region, a stabilization of actin-fibers in the distal region, and an inhibition of pollen tube growth by 80% [88].

#### Actin self-assembly and contractility of the actomyosin cytoskeleton

The self-assembly of actin is also pH-dependent. In nematode spermatozoa, faster assembly and lower G-actin concentrations occur at lower pH values [177], such as those found in the rear end of migrating cells [95]. In addition, the contractility of reconstituted active actin systems is tightly controlled by local pH [74]. Contractility increases as pH decreases. This is due to the fact that the intrinsic cross-bridge strength of myosin-II is pH-dependent and thus accountable for a sharp transition of the actin/myosin-II activity from *non-contractile* to *contractile* by a pH change of not more than 0.1 [74]. Consequently, a cytosolic pH gradient of up to 0.2 pH units [95] allows cell rear retraction in migrating cells while maintaining a flow with strongly reduced overall contractility at the protruding cell front.

Interestingly, pH_i_ also precisely controls the assembly of the unique major sperm protein (MSP) filament system of migrating nematode sperm cells. The pseudopodium exhibits a pH_i_ gradient with pH 0.15 units higher at the leading edge, where fiber complexes assemble, than at the base, where disassembly takes place [70].

#### Cdc42

The small GTPases Rac, Rho, and Cdc42 are hierarchically linked to each other and play key roles in both establishing cell polarity and directional cell migration [92, 132]. The cell front contains higher active Rac and lower active Rho concentrations, while the rear end has lower active Rac and higher active Rho concentrations. Cdc42 is recruited to the leading edge where a guanine nucleotide-exchanger factor (GEF) catalyzes GTP binding by Cdc42. GEF binding to phosphoinositol 4,5-bisphosphate is pH-dependent and, as shown in fibroblasts, requires H^+^ efflux by NHE1 activity [39]. For RasGRP1, a Ras-specific GEF, His^212^ has been identified as a pH sensor that activates RasGRP1 when the inside of the cell becomes less acidic. When the charge on His^212^ changes from positive (protonated) to neutral (deprotonated), the RasGRP1 protein opens up to bind to DAG (diacylglycerine) at the membrane [174].

## Molecular pH sensors: basic and acidic amino acids

The previous paragraphs insinuate that over the last two decades (de)protonable basic and acidic amino acids, particularly histidine and aspartate, have emerged as molecular pH sensors that translate pH changes into conformational changes of proteins and thus affect their catalytic activity, substrate binding, stability, interaction, aggregation, and localization [151]. The protonation of proteins can be considered a reversible posttranslational modification, analogous to phosphorylation, methylation, and ubiquitination. This proton-mediated posttranslational modification represents a certain signaling specificity as it applies to only a minority of sites in selective proteins that titrate within the physiological pH range [135]. Relevant examples in addition to the above-mentioned are the epidermal growth factor receptor (EGFR), the transcription factor p53, the signaling and adherens junction protein β-catenin [184, 185], and the proton-sensing G protein–coupled receptors OGR1 and GPR4 [91].

EGFR exhibits pH-sensitive kinase activity and confers increased pathway activation at higher pH_i_ when arginine at position 776 is mutated to histidine. This results in increased proliferation, accelerated malignant transformation [185], and possibly the acquisition of migratory capabilities as typical of cancer cells with higher pH_i_ values.

Similarly, p53 shows a pH-sensitive transcriptional activity when displaying the recurrent somatic mutation from arginine^273^ to histidine [59, 185]. The transcriptional activity and cell death in response to DNA damage are decreased at higher pH_i_.

With regard to cell motility, β-catenin is of interest for various reasons. Activation of β-catenin down-regulates cell–cell junction–related genes, induces epithelial-to-mesenchymal transition [68], regulates genes implicated in migration such as paxillin [2], and stabilizes the front-rear polarity of migrating cells [173]. Degradation of β-catenin needs phosphorylation of N-terminal residues for recognition by the E3 ligase β-TrCP. Not the phosphorylation step itself but the concomitant binding to β-TrCP depends on pH. A higher pH_i_ induces increased β-TrCP binding and thus decreases β-catenin stability [184]. Interestingly, in contrast to EGFR or p53, it is not a mutation of arginine to histidine, but a somatic mutation of His^36^ to arginine [37] that allows β-catenin to bypass pH sensitivity, i.e., the pH-sensitive β-TrCP recognition (and degradation), which then results in increased Wnt pathway activity in cancer cells [14].

pH_e_ and its changes can be detected and transduced by the proton-sensing G protein–coupled receptors OGR1 and GPR4 [91]. While OGR1 signals via inositol phosphates (IP), GPR4 elicits cAMP signaling. Both IPs and cAMP are central players in cell migration [55, 56, 168] and reach their maximum production at pH_e_ 6.8. Five histidines (His^17, 20, 84, 169, 269^), including the interactions of His^17^ with His^84^ and His^20^ with His^269^, allow for the pH_e_-dependent OGR1-mediated IP- and GPR4-mediated cAMP production [91].

In the end, being one of the major regulators of cellular pH homeostasis, even NHE1 itself displays a cluster of histidine residues in the proximal C-terminal cytoplasmic domain. This histidine cluster regulates pH-dependent PI(4, 5)P_2_ binding and transporter activity [181].

## pH taxis—directional migration along a pH gradient

When the extracellular bulk pH_e_ is in the form of a gradient, it can serve as a directional stimulus. Such gradients are found within tumors, in cutaneous wounds, and at inflammatory sites.

At the interface between human prostate tumors grown in mice and the surrounding normal tissue, pH_e_ increases about 0.4 units (~ 6.9 to ~ 7.3) over 1 mm towards the normal tissue [41], and within human colon adenocarcinoma xenografts, pH_e_ decreases by about 0.7 units (~ 7.4 to ~ 6.7) over ~ 350 nm from a tumor blood vessel [52]. Although hardly measured explicitly in vivo in the human body, the presence of such gradients is highly probable in a number of solid human tumors in situ. The nanoprobe ONM-100, which emits fluorescence at pH values below 6.9 while its fluorescence is quenched at pH values above 6.9, has been successfully applied to identify breast, esophageal, and colorectal tumors, and squamous cell carcinomas in the head and neck region, prior to surgical resection. The images used to localize these tumors actually imply centrifugal fluorescence gradients within the cancer tissue [84, 175].

In chronic cutaneous wounds, pH_e_ increases considerably from the wound edge (below 6.5) to the wound center (above 7.4) over distances of less than 1 cm [136].

At inflammatory sites, e.g., during the course of inflammatory responses against bacteria in peripheral tissues, pH_e_ can be as low as 5.5. This drop in pH_e_ can be caused by (i) a shift to glycolytic metabolism in response to tissue hypoxia resulting from the damage of small blood vessels and the metabolic activity of infiltrating leukocytes, (ii) a massive production of protons by neutrophils during the activation of the respiratory burst, and (iii) the accumulation of short-chain fatty acids produced by bacteria [32].

**Fig. 4:**
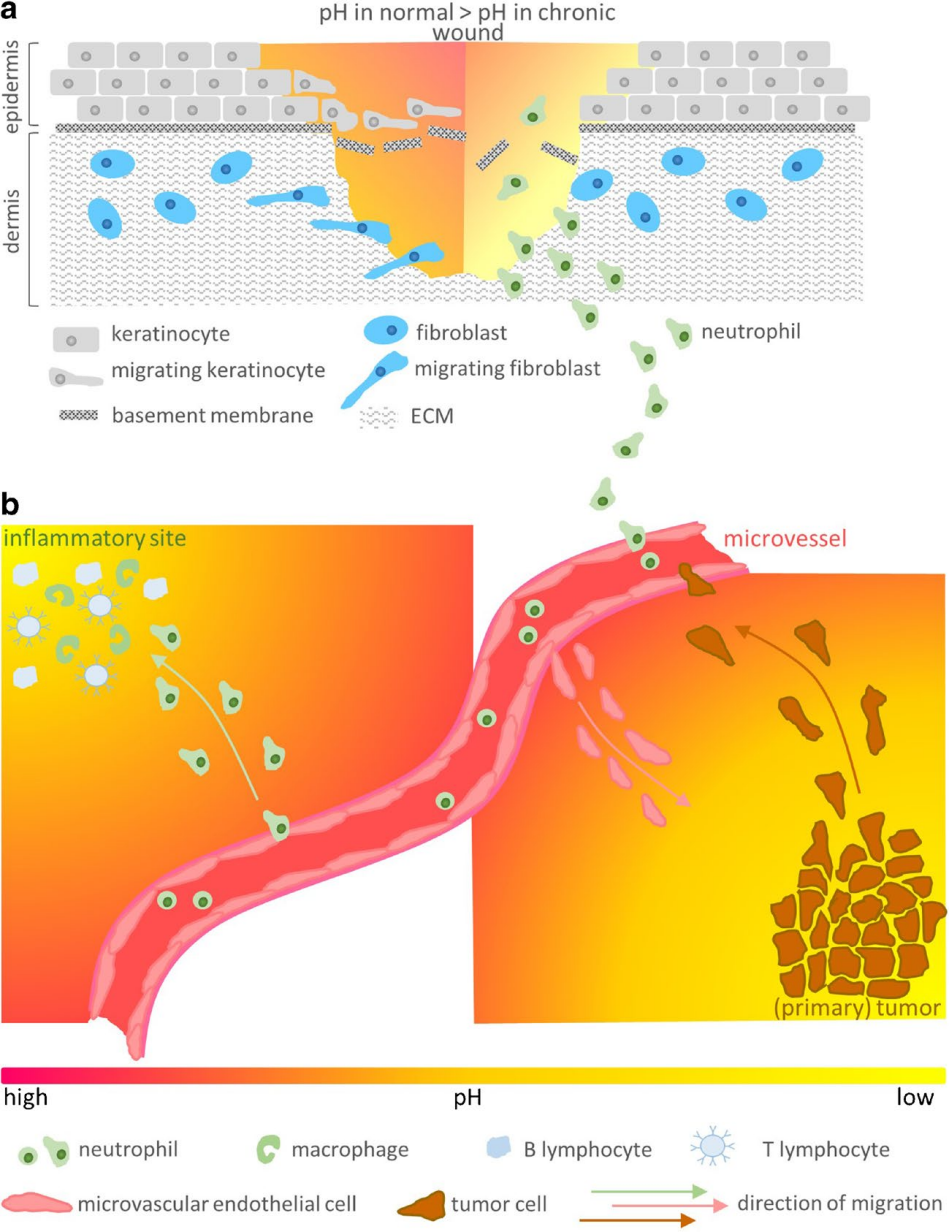
Directional cell migration along pH gradients. **a** In a normal wound (left half), epidermal keratinocytes and dermal fibroblasts migrate towards the more alkaline pH_e_ in the center of the wound in order to close it. In a chronic wound (right half), a quite acidic pH at the wound margin prevents keratinocytes and fibroblasts from migrating into the wound. Instead, neutrophils are attracted by the low pH. Once inside the wound area, their presence including the local secretion of inflammatory mediators fuels the inflammatory process and thus prevents wound healing. **b** In general, neutrophils migrate towards inflammatory sites that typically are acidic (left). Microvascular endothelial cells also migrate to tissue areas with an acidic pH value, while metastatic tumor cells migrate away from the acidic tumor tissue to regions with a physiological pH value near blood and lymph vessels. Please see the section on pH taxis for further details and references

Depending on the cell type and the physiological context, cells migrate either towards or away from the lower pH (Fig. 4b). Chinese hamster ovary (CHO) cells engineered to express α_v_β_3_ integrin and primary bovine retinal microvascular endothelial cells (MVECs, endogenously expressing α_v_β_3_ integrin), both seeded on fibronectin, migrate towards the acidic end of a pH_e_ gradient, ranging from pH 7.5 to 6.0, due to a preferential polarization towards the lower pH [114]. The migration velocity is steadily decreasing as cells move towards the lower pH, confirming that there is an optimum pH for migratory activity as shown for human melanoma (MV3) cells [155]. Integrin activation including outside-in signaling in response to acidic pH_e_ may represent one of the major potential mechanisms underlying pH taxis. In this way, acidic pH_e_, as found in hypoxic tissue, could stimulate MVEC migration and angiogenesis in order to ensure sufficient vascularization and oxygen and nutrient supply. However, it has not been clarified yet to what extent mere pH-dependent cell–matrix and cell–cell interactions, including integrin-mediated downstream signaling, can fulfill the task of a pH detector. It can be almost certainly ruled out that this mechanism plays an exclusive role in pH detection. Accordingly, as for endothelial cells, acidic pH was found to downregulate vascular endothelial growth factor receptor-2 (VEGF-2) and thus impede VEGF-mediated migration [34]. In the latter study, however, the experimental set up did not include a pH_e_ gradient, but instead the cells were exposed to a homogenous bulk pH, and furthermore, both EC sprouting and tubulogenesis remained unaffected by extracellular acidity.

Human metastatic breast cancer cells (MDA-MB-231 cells) seeded on type I collagen migrate in the direction of higher pH_e_ values in a gradient of 0.2–0.3 pH units/mm [166]. The slope of this gradient would correspond to a gradient of 0.02 units per single cell length and thus would only be a tenth of the NHE1-generated intra- and pericellular pH gradients in MV3 cells [95, 157]. Given the observations that in MV3 cells (i) the pH gradient at the cell surface is stabilized by the glycocalyx [77], and (ii) the protons extruded by polarized distributed NHE1 have a stronger impact on migration than those present in the bulk solution [161], it is difficult to visualize a mechanism by which an externally applied pH_e_ gradient of 0.02 units could prevail the intrinsic, pericellular pH gradient of 0.2 units. The question of how the shallow ambient pH_e_ gradient modifies the steeper pH gradient in immediate proximity to the cell surface in order to either enhance or mitigate directed cell migration has remained unanswered to date. An involvement of both the glycocalyx as a cell surface–buffering compartment accepting/releasing protons [77, 157] and proton-sensing G protein–coupled receptors [159, 183] seems possible. pH-dependent MMP activity (please be referred to the above paragraph on cell invasion) and the pH-dependent properties of substrate components including pH-dependent steps of ECM genesis such as collagen fibrillogenesis [51, 76, 82], fiber crosslinking [23], or self-assembly of laminin [40] should be kept in mind as well. Independently of the underlying mechanism, for a cancer cell to intravasate and metastasize, it is essential to move away from the acidic primary tumor towards the most alkaline regions surrounding blood and lymph vessels (Fig. 4b).

In chronic cutaneous wounds, pH_e_ rises with increasing distance from the wound edges. The strongly acidic margins of the wound (pH ≤ 6.5) prevent healing by reducing the viability, proliferation, and migration velocity of keratinocytes [78, 136]. Thus, the centripetal keratinocyte recruitment along the pH_e_ gradient from the acid wound periphery towards the more alkaline wound center (pH ≥ 7.4) is critically low (Fig. 4a). Of note, this pH_e_ gradient mirrors the wound’s NHE1 expression profile that shows a centrifugally rising NHE1 expression (136). Simply increasing pH_e_ from below 6.5 to 6.8 (i) enables keratinocytes to migrate directionally towards the higher pH_e_ in the wound center [136], (ii) increases wound closure in an in vitro fibroblast wound healing assay, and (iii) accelerates re-epithelialization [78]. pH-sensing G protein–coupled receptors are thought to play a role not only in tumor cell migration and metastasis but also in the wound healing process [183]. To what extent proton-sensing G protein–coupled receptors contribute to the detection of an external pH_e_ gradient and pH tactic behavior is yet to be investigated in detail. In consideration of the chronic wound’s characteristic pH profile, both modulating pH_e_ by applying pH-restoring hydrogel-like dressings [75] and the application of pH_e_ responsive wound dressings have been recognized as a potential therapeutic strategy [50, 103].

Superimposition of a chemical and a pH_e_ gradient, as found at sites of inflammation, most efficiently stimulates NHE1-dependent, Cdc42-mediated directional migration of neutrophils [111]. Formylated peptides and complement molecule C5a, both secreted at the site of inflammation, induce neutrophil chemotaxis. Even in the absence of these chemoattractants, neutrophils tend to migrate from pH_e_ 7.5 towards lower pH values, however lose their directionality at pH_e_ 7.2 already. In the presence of a C5a gradient, an additionally superimposed pH_e_ gradient supports neutrophil chemotaxis in the direction of the C5a source and the lower pH_e_. While directionality is strongly inhibitable with the NHE1 inhibitor cariporide and steadily decreases towards pH_e_ values as low as 6.5, the migration velocity itself remains nearly unaffected by pH_e_ and is comparably less affected by cariporide, suggesting that the steering mechanism (chemotaxis) rather than the migration motor (velocity) depends on proper ambient pH and NHE1 activity. In addition to pH_e_, the intermediate chemoattractant leukotriene B_4_ (LTB_4_) is required for efficient chemotaxis towards the end-target chemoattractant C5a. The secretion of LTB_4_ from activated neutrophils also depends on NHE1 activity and pH_i_ and thus indirectly on pH_e_. It decreases with falling pH_e_ and pH_i_ or when NHE1 activity is inhibited [111]. NHE1 activity as a requirement for chemotaxis has been shown also for polymorphonuclear leukocytes in an fMLP gradient [127]. In summary, a shallow proton gradient with pH_e_ values decreasing from blood vessels towards the inflamed tissue may accelerate extravasation of stimulated neutrophils and then direct them to the inflammatory site (Fig. 4b). Once in the acidic inflammatory microenvironment, neutrophil chemotaxis is impaired while migration velocity is only slightly reduced, allowing cells to ramble on-site through the inflamed tissue.

In quite a number of cell types, NHE1 turns out to be essential for directional migration although other acid/base regulating transporters such as Na^+^-HCO_3_^−^ cotransporters (NBCs) are also present in the plasma membrane. This holds true even in the absence of a directional stimulus as shown for Madin-Darby canine kidney (MDCK) cells. Whereas Na^+^-HCO_3_^−^ co-imported by NBC1 can compensate for a lack in NHE1 activity with respect to cellular pH homeostasis and volume regulation, in the absence of a functional NHE1, NBC1 does not provide the cell polarity required for directional cell migration [140]. This mechanistic predominance of NHE1 over NBCs reduces the dependence on the CO_2_/HCO_3_^−^ carried fraction of the extracellular buffer capacity. This could be particularly important under in vivo conditions, where the buffering capacity of the open CO_2_/HCO_3_^−^ system can vary considerably depending on CO_2_ production in the tissue relative to its removal, e.g., by the blood stream. Thus, the dominance of NHE1 over NBCs could ensure continuous and smooth directional migration in the face of a potentially unstable extracellular HCO_3_^−^ concentration. Furthermore, it emphasizes the relevance of a tightly controlled pH profile directly at the cell surface. The pH nanodomains locally generated by NHE1 [90] and its cytoskeletal anchoring, both affecting adhesion, related signaling events, and cytoskeletal dynamics, are virtually certain to regulate asymmetric signals that establish polarity and a differential, coordinated focal adhesion remodeling at the cell front and rear [30]. However, what is the underlying mechanism that would master NHE1 targeting, that is, how is directional trafficking of NHE1 towards the cell front regulated, and how is the cell front as such established in the first place? A redistribution of randomly distributed NHE1, accompanied by the formation of a polarized morphology and the acquisition of the ability to migrate, has been observed in cervical cancer cells upon exposure to EGF [20]. Nevertheless, the mechanism behind the directed trafficking of NHE1 in single cells, especially in the absence of any directional stimulus, remains unexplained to date and needs further examination.

## Does pH contribute to self-guidance mechanisms?

The fact that migrating cells themselves generate directional information via a dynamic interplay of cell-intrinsic and cell-extrinsic regulators and thus have more control over their directionality than previously assumed has been reviewed recently [188]. In terms of single cell migration, there are three different self-guidance mechanisms [188], and pH strongly affects all three of them.

First, “subcellular symmetry breaking” is based on intracellular traveling waves of the cytoskeleton and signaling events via the phosphatidylinositol-4.5-bisphosphate system [7, 43]. The resulting break in the symmetry of the cell cortex then defines sites of cellular protrusions and lamellipodial outgrowth [100]. This symmetry breaking includes the above-mentioned pH-dependent cytoskeletal dynamics and its pH-dependent components gelsolin, ADFs/cofilin, actin self-assembly, actomyosin contractility, and also the small GTPase Cdc42. Hence, the intracellular pH gradient along the direction of movement with higher pH_i_ values at the cell front or in protrusions, e.g., invadopodia, and lower pH_i_ values towards the rear end [90, 95] most likely contributes to the perpetuation of “subcellular symmetry breaking,” if not even its generation.

Second, during “self-generated chemotaxis,” cells create their own local, dynamic gradients by breaking down, sequestering, or scavenging surrounding attractant molecules [110, 133]. In this way, a given, yet non-detectable gradient can be amplified in the immediate vicinity of the cells and thus becomes detectable. Normally, chemotactic cells identify attractant gradients by comparing attractant receptor occupancy between their front and rears. Differences as low as 1% can be distinguished. However, beyond distances of 0.5 up to 1 mm, the given gradients contain zones that would be either too saturating or too shallow to cause the detectable 1% occupancy difference [169]. At this point, the amplification mechanism comes into play to promote long-range chemotaxis and even allow cells to navigate mazes [170]. An almost perfect example for the role of acid extruders and pH_e_ in “self-generated chemotaxis” is the above-mentioned NHE1, pH_e_, pH_i_, and Cdc42 dependence of directional neutrophil migration [111].

Third, cells create “self-organized extracellular scaffolds” by exerting forces towards the ECM. The resulting changes in the ECM’s physical properties and its structure, such as either parallelizing or disarranging ECM fibers, impact stiffness, order, topology, and porosity, and can then act as guidance cues to drive directional cell migration [22, 24, 172]. The strength of α_2_β_1_ integrin–mediated adhesions to collagen type I depends on cell surface pH, and at pH_e_ 6.8 or when NHE1 activity is stimulated by intracellular acidification with propionic acid, human melanoma (MV3) cells pull on ECM fibers potentially rearranging them [155]. In addition to adhesion-affected re-arrangement of the matrix, local digestion of the ECM by pH- and thus NHE1-dependent MMP activity contributes to “self-organized extracellular scaffolds” as well [16, 47, 154, 171].

In conclusion, once the polarized distribution of (net) acid extruders such as NHEs or NBCs is established, their activity may perpetuate pH_i_ and pH_e_ gradients along the direction of movement. A concerted action of pH-regulating and pH-dependent proteins as well as locally controlled osmotic swelling and shrinkage [139] may generate an intrinsically self-sustaining process making one think of a perpetual motion machine. Hence, pH does have an essential share in self-guidance. To what extent direct pH sensing by lipids in membranes [3] and subsequent signaling contributes to keeping the migration machinery going is quite an appealing question that certainly demands further investigation.

## Acid priming

Several cell types, even metabolically highly active tumor cells with generally upregulated net-acid extruding transporters compared with normal cells [42], e.g., human melanoma [161], lung carcinoma, and normal bronchial epithelial cells [146], rat prostate carcinoma cells [124], or neutrophils [67, 111], respond to acidic pH_e_ exposure by lowering their pH_i_ values. Assuming that the intracellular acidification is a direct consequence of the extracellular acidification because (net) acid extruders such as NHEs and NBCs cannot work efficiently enough against the increasing extracellular proton concentration, the intracellular protons may act as messengers via cytosolic proteins with integrated molecular proton sensors, predominantly histidine and aspartate residues (see above). This procedural sequence would not require the presence of proton-sensing G protein–coupled receptors in the plasma membrane, but may be prone to interfering effects caused by pH-sensitive cation channels. As reviewed by Pethö et al. [115], acidic pH_e_ stimulates Ca^2+^ (TRPV1; TRPC4, 5; P_2_X_1–4_) and Na^+^ (ASICs) conducting channels. While the resulting Na^+^ influx and the concomitant changes in the Na^+^ gradient across the plasma membrane may affect the activity of Na^+^-dependent acid/base regulators and consequently pH_i_, a rise in [Ca^2+^]_i_ can elicit Ca^2+^ signaling, e.g., via Ca^2+^/calmodulin-dependent protein kinases, and thus stimulate migration. However, in pancreatic stellate cells (PSCs) exposed to pH_e_ 6.6 overnight, [Ca^2+^]_i_ is significantly lower, [Na^+^]_i_ significantly higher, and the membrane potential strongly hyperpolarized compared to cells kept at pH_e_ 7.4 which impels the Na^+^/Ca^2+^ exchanger NCX1 that usually removes Ca^2+^ from the cytosol to function in reverse mode. According to this, NCX1 inhibition stimulates PSC migration at pH_e_ 7.4 because Ca^2+^ ions are retained in the cytosol, whereas it abates migration at pH_e_ 6.6 due to a blocked Ca^2+^ entry [85].

On the other hand, Ca^2+^ signaling is modulated by pH_i_. Due to slow diffusion and buffer sharing, alterations in pH_i_ and [Ca^2+^]_i_ can be compartmentalized. Free Ca^2+^ ions and free protons compete for binding to the same cytoplasmic Ca^2+^/H^+^ buffer molecules with sufficient mobility, i.e., low molecular weight, such as histidyl peptides, e.g., carnosine, and ATP [165]. In this way, Ca^2+^ bound to such a buffer molecule can be recruited uphill from anywhere in the cytosol to localized acidic nanodomains where it is then released. This spatial Ca^2+^/H^+^ coupling is likely to be of general importance in local cell signaling [165]. Such a Ca^2+^/H^+^ coupling could also explain the, probably Ca^2+^/calmodulin-mediated, activation of PI3K in cells whose migration is stimulated by an acidic environment without the involvement of acid-sensing ion channels (ASICs), e.g., glioblastoma cells or neutrophils [25, 97].

Long-term exposure to an acidic environment can initiate long-lasting effects including an aggressive migratory behavior observable even hours after the cells’ return to physiological pH values. This acidic priming with “memory effect” is a matter of particular interest because it enhances the metastatic potential of cancer cells by allowing for malignant behavior in a physiological environment far away from the primary tumor [123]. In this context, Thews and Riemann [167] have recently reviewed the various outside-in signaling pathways induced by acid exposure and the signaling effects mediated by intracellular acidification. A low pH_i_ resulting from extracellular acidification causes the release of reactive oxygen species (ROS) from mitochondria. ROS induces MAPK activation and stimulates gene expression via p38 and the transcription factor CREB. The increased transcriptional activity of CREB can persist for 24 h after returning the cells back to normal pH [124]. However, while the acidosis-induced increase in ROS seems imperative for an increased motility of rat prostate cancer cells, the augmented phosphorylation of ERK1/2 and p38 is not required [123]. Nevertheless, 48 h exposure of human triple-negative breast cancer (MDA-MB-231) and mouse mammary carcinoma (4T1) cells to pH_e_ 6.4 caused changes in the expression and splicing of 2752 genes, including a number of those affecting cell motility, potentially controlled through a specific set of RNA binding proteins and downstream of pH-induced chromatin modifications [128]. Similarly, in human pancreatic ductal adenocarcinoma cell lines, long-term (1-month) acidic pressure (pH_e_ 6.6) selects cells with enhanced migration and invasion abilities induced by epithelial-mesenchymal transition (EMT), intensifying their metastatic potential when re-exposed to pH_e_ 7.4, and in acid-selected human pancreatic ductal adenocarcinoma (PANC-1) cells, genes relevant to proliferation, migration, EMT, and invasion are upregulated [4].

In summary, acid exposure can bring about a “memory effect” that is induced by pH-affected signaling including ROS production and accompanied by transcriptome rewiring. This enables particularly tumor cells to acquire a more malignant and metastatic phenotype in an acidic environment such as primary tumor tissue and to then maintain a pronounced migratory and invasive behavior for several hours up to even more than a day after leaving the acidic tissue.

## Conclusion

Both pH_e_ and pH_i_ have a hand in single cell migration. pH_e_ strongly affects cell adhesion and cell morphology, and under optimum conditions supports the establishment of a polarized morphology, a prerequisite for migration. pH_i_ has an impact on a number of focal adhesion components, actin polymerization, and actomyosin contractility. There are considerable differences between species, cell types, and cell lines in terms of pH-dependent morphology, optimum adhesion, migration and invasion, and the direction of pH taxis. The set of expressed adhesion molecules alone can make a difference, because different integrin-matrix interactions exhibit different pH optima. Human melanoma (MV3) cells express α_2_β_1_ integrins that mediate optimum cell migration on collagen type I at a bulk pH_e_ of 7.0–7.2 and basal NHE1 activity producing a cell surface pH of 7.2–7.4 at the leading edge [90, 157]. In comparison, in human breast cancer (MCF-7) cells expressing both α_2_β_1_ integrins and a constitutively active receptor tyrosine kinase (ErbB2), cell surface pH is 7.05, and only an increase to 7.2 by means of NHE1 inhibition leads to optimum migration on collagen I [80]. This confirms a cell surface pH of ~ 7.2 to be optimal for α_2_β_1_ integrin–mediated cell migration, independently of the cell type. Thus, not in spite of, but because of the species- and cell type-dependent differences in the pH characteristics of motility parameters, it is tempting to see the function of pH/protons in the regulation of cell motility to be a widespread if not even cross-species universal mechanism. Given (i) the multitude of pH-sensitive molecules functioning as adjustable setscrews of the migration machinery, (ii) their mutual, pH-dependent interactions, and (iii) the pH-sensitive activity of MMPs, protons are modulators of migration and drivers of invasion, and, therefore, can be considered intra- and intercellular messengers. Mechanistically, (de)protonation of acidic and basic amino acids, particularly histidine and aspartate, translates pH into catalytic activity, substrate binding, stability, interaction, aggregation, and localization of proteins.

An interesting aspect in relation to therapeutic strategies is the extracellular pH as an adjustable screw that could be taken advantage of in order to accelerate wound healing, control inflammatory processes, and reduce invasion and metastasis. This implies that the molecular mechanisms underlying pH-regulated cell migration, which harbor great potential as therapeutic targets, need to be further investigated. In this context, the proton-sensing GPCRs, ASICs, and the proton-sensing members TRPV1 and TRPV4 from the transient receptor potential channel vanilloid subfamily certainly deserve further in-depth consideration.

## Data Availability

This declaration is not applicable
