# Correction to: pH‑regulated single cell migration

**DOI:** 10.1007/s00424-024-02970-9

**Published:** 2024-05-08

**Authors:** Christian Stock

**Affiliations:** Department of Gastroenterology, Hepatology, Infectiology & Endocrinology, Hannover Medical School, Carl‑Neuberg‑Str. 1, 30625 Hannover, Germany

**Correction to** **:** **Pflügers Archiv - European Journal of Physiology (2024) 476:639–658**

10.1007/s00424-024-02907-2

In the originally published article, on page 641 in the right-hand main text column below Figure 1, the decimal points of the three numerical values given for intracellular and extracellular pH gradients were erroneously shifted to the right by one decimal place, so that the specified pH values were considerably too high.

The actual values are: (i) a ΔpH of 0.15 for the cytosolic pH difference between the more alkaline lamellipodium and the more acidic posterior end in human and murine melanoma cells, (ii) a ΔpH of ~0.05 for the cytosolic pH gradient in non-malignant cells, and (iii) a ΔpH of up to 0.2 for the cell surface pH gradient between the more acidic leading edge and the rear end of MV3 cells.

The original article has been corrected.

